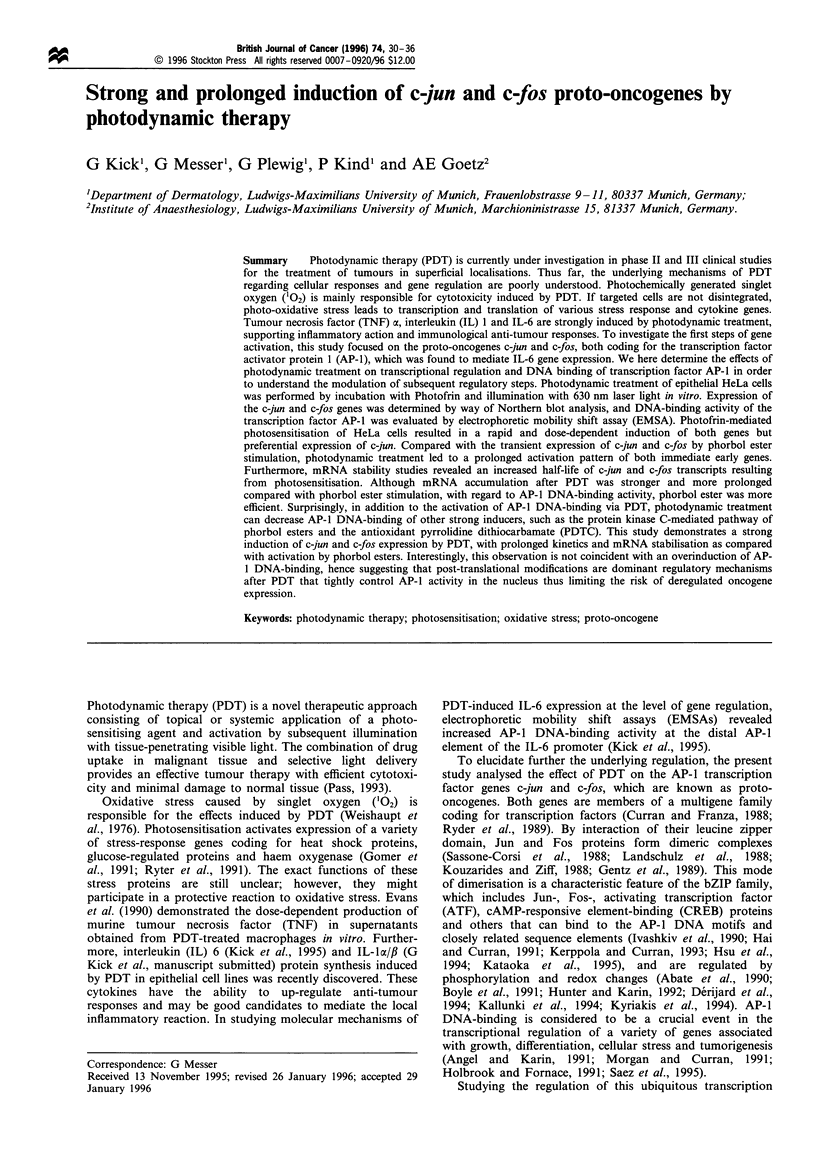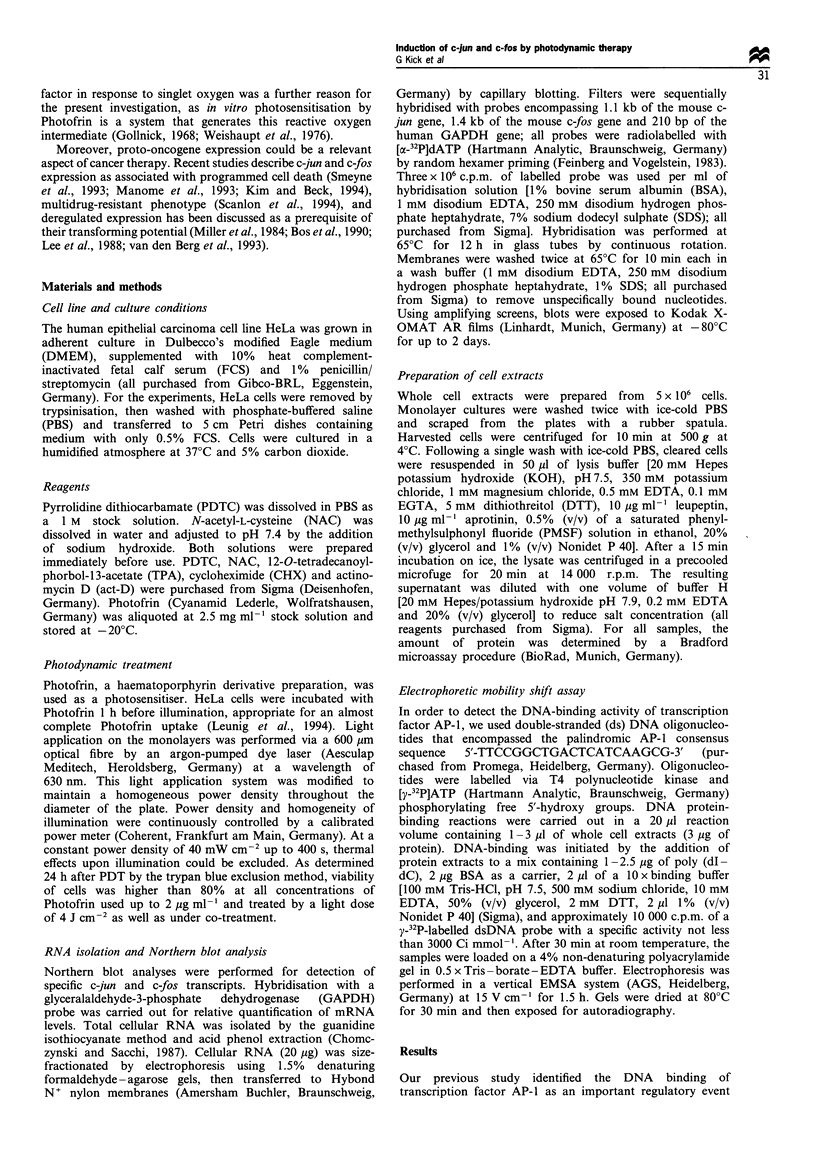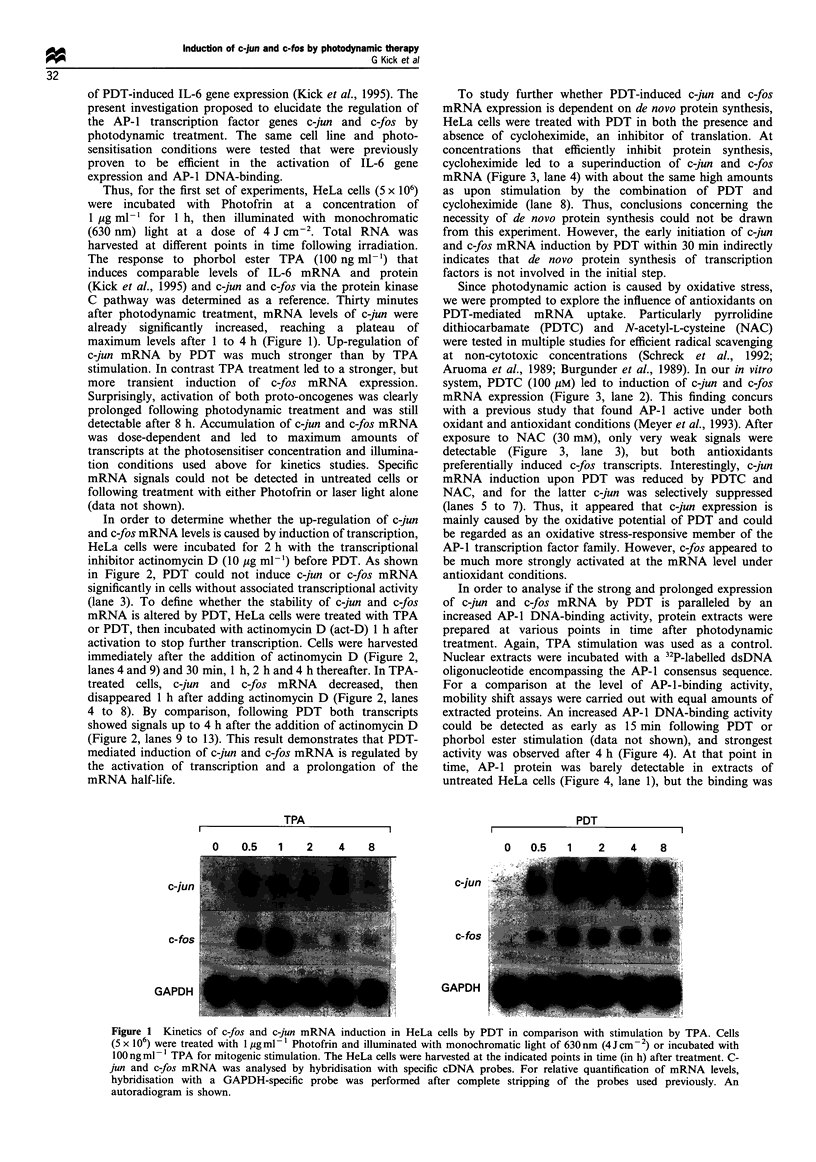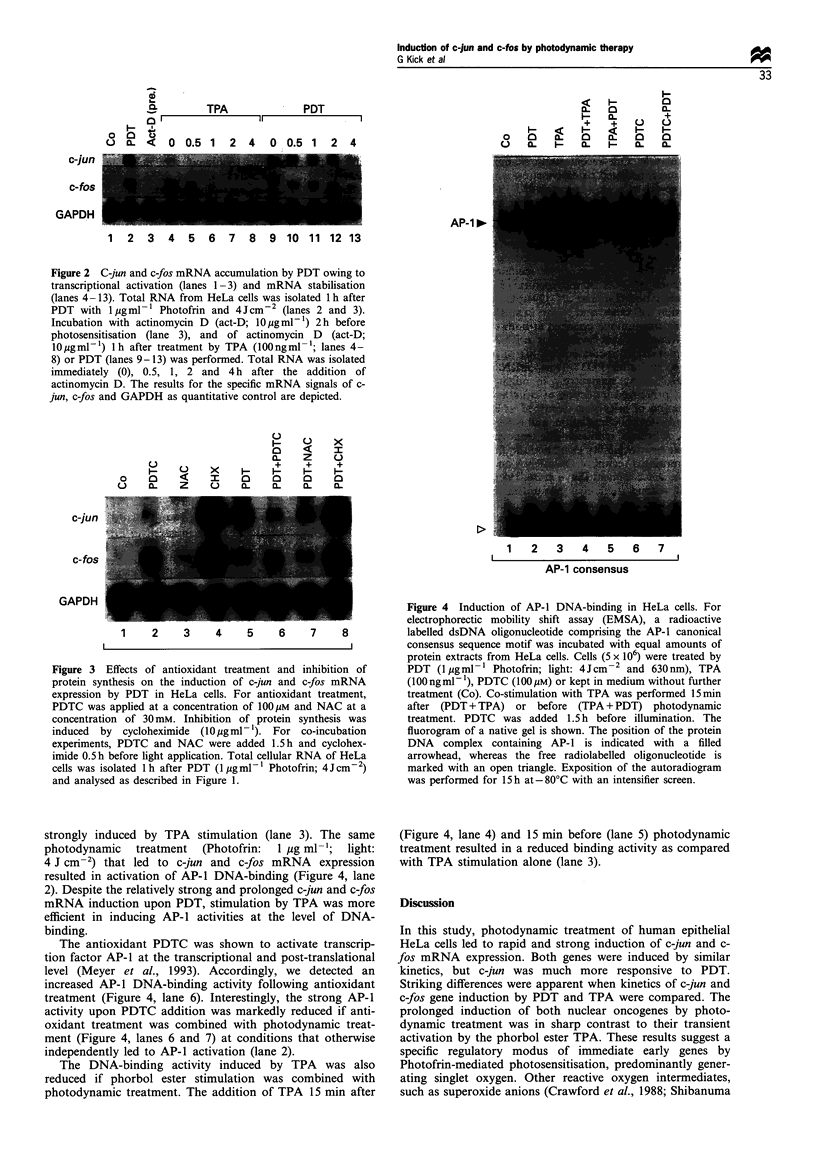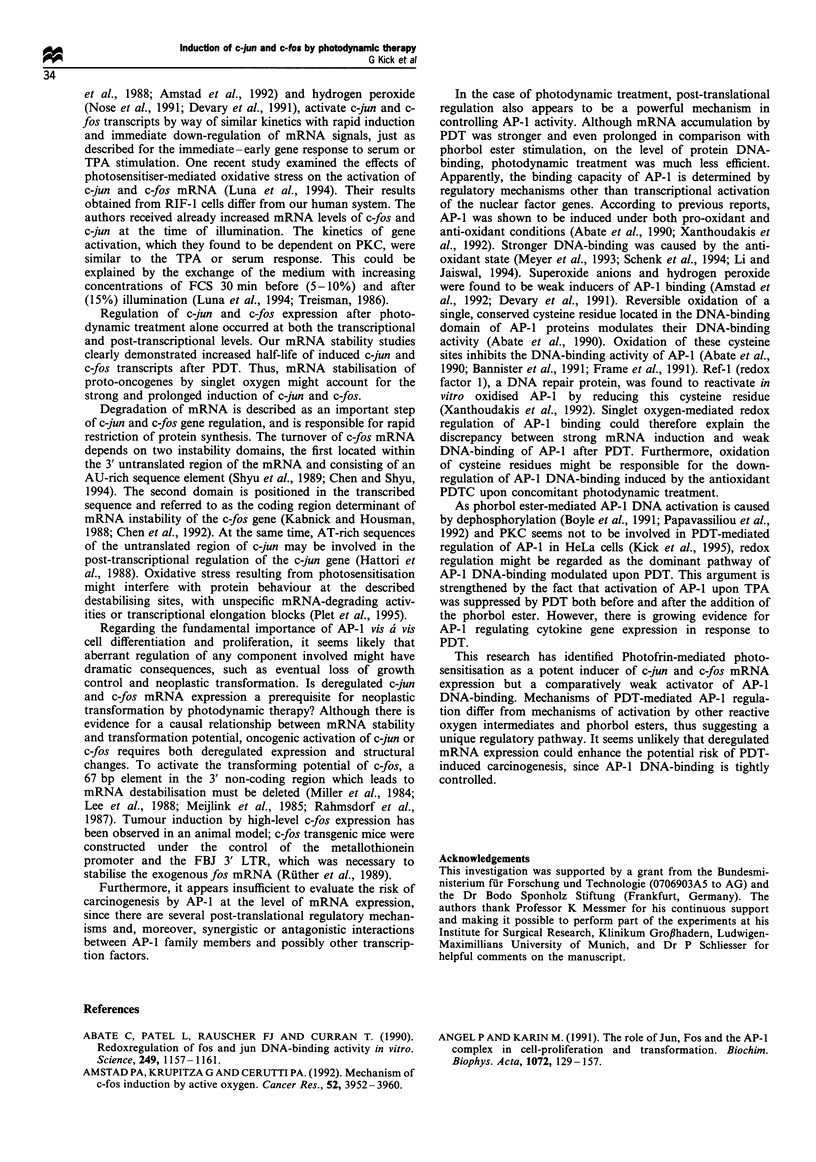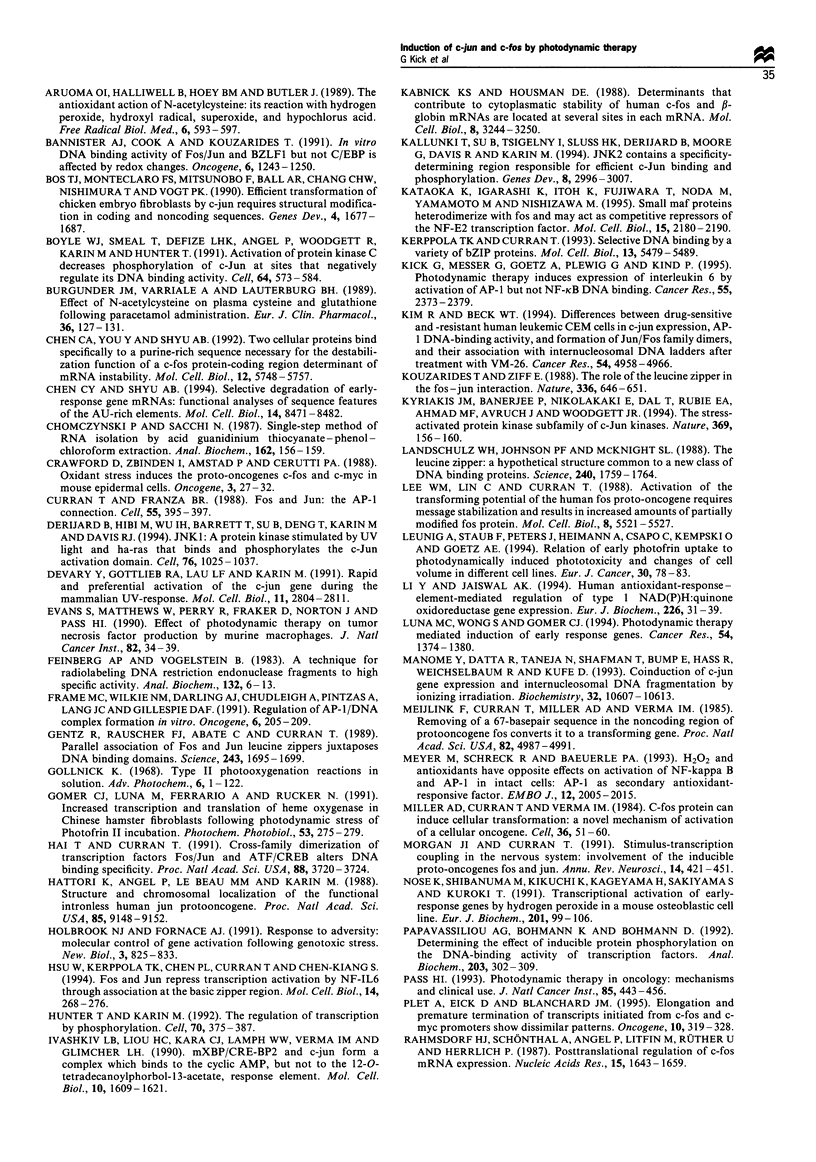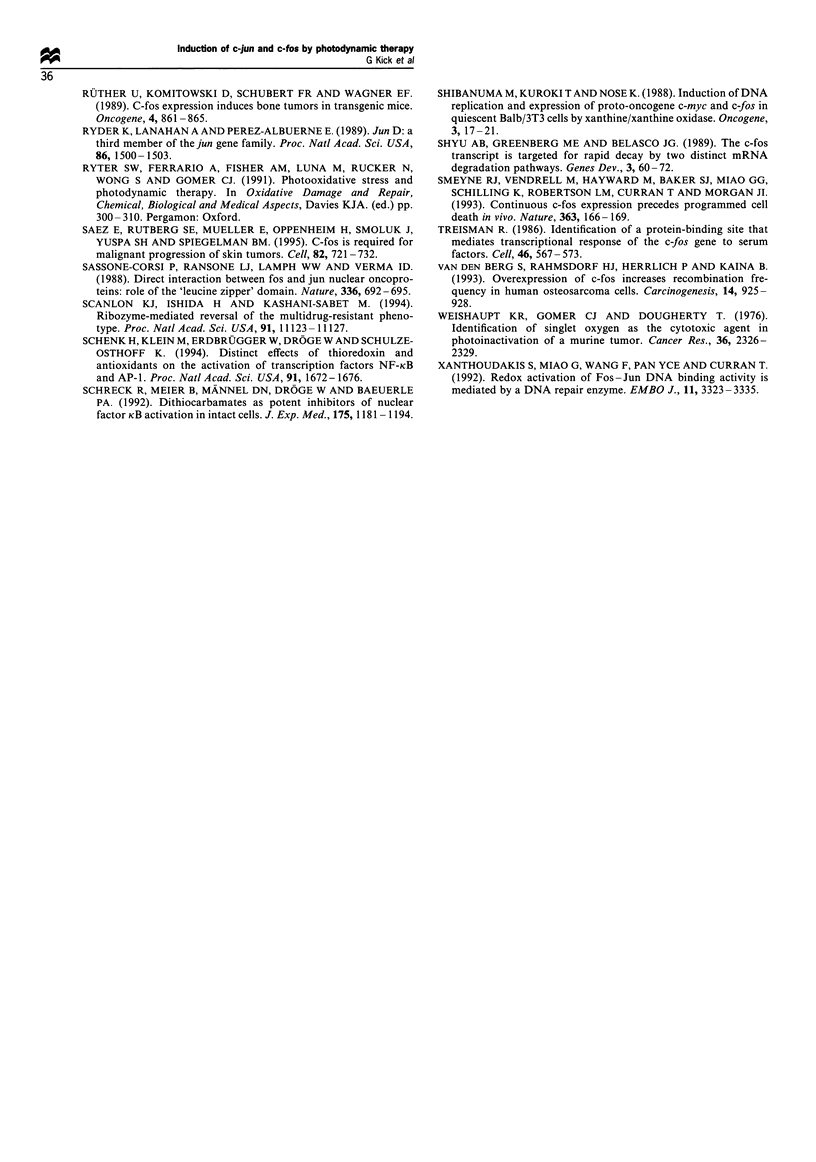# Strong and prolonged induction of c-jun and c-fos proto-oncogenes by photodynamic therapy.

**DOI:** 10.1038/bjc.1996.311

**Published:** 1996-07

**Authors:** G. Kick, G. Messer, G. Plewig, P. Kind, A. E. Goetz

**Affiliations:** Department of Dermatology, Ludwigs-Maximilians University of Munich, Germany.

## Abstract

**Images:**


British Journal of Cancer (1996) 74, 30-36
© 1996 Stockton Press All rights reserved 0007-0920/96 $12.00

### Strong and prolonged induction of c-*jun* and c-*fos* proto-oncogenes by photodynamic therapy

G Kick[1], G Messer[1], G Plewig[1], P Kind[1] and AE Goetz[2]

[1]*Department of Dermatology, Ludwigs-Maximilians University of Munich, Frauenlobstrasse 9-11, 80337 Munich, Germany;* [2]*Institute of Anaesthesiology, Ludwigs-Maximilians University of Munich, Marchioninistrasse 15, 81337 Munich, Germany.*

**Summary** Photodynamic therapy (PDT) is currently under investigation in phase II and III clinical studies for the treatment of tumours in superficial localisations. Thus far, the underlying mechanisms of PDT regarding cellular responses and gene regulation are poorly understood. Photochemically generated singlet oxygen ($^1O_2$) is mainly responsible for cytotoxicity induced by PDT. If targeted cells are not disintegrated, photo-oxidative stress leads to transcription and translation of various stress response and cytokine genes. Tumour necrosis factor (TNF) α, interleukin (IL) 1 and IL-6 are strongly induced by photodynamic treatment, supporting inflammatory action and immunological anti-tumour responses. To investigate the first steps of gene activation, this study focused on the proto-oncogenes c-*jun* and c-*fos*, both coding for the transcription factor activator protein 1 (AP-1), which was found to mediate IL-6 gene expression. We here determine the effects of photodynamic treatment on transcriptional regulation and DNA binding of transcription factor AP-1 in order to understand the modulation of subsequent regulatory steps. Photodynamic treatment of epithelial HeLa cells was performed by incubation with Photofrin and illumination with 630 nm laser light *in vitro*. Expression of the c-*jun* and c-*fos* genes was determined by way of Northern blot analysis, and DNA-binding activity of the transcription factor AP-1 was evaluated by electrophoretic mobility shift assay (EMSA). Photofrin-mediated photosensitisation of HeLa cells resulted in a rapid and dose-dependent induction of both genes but preferential expression of c-*jun*. Compared with the transient expression of c-*jun* and c-*fos* by phorbol ester stimulation, photodynamic treatment led to a prolonged activation pattern of both immediate early genes. Furthermore, mRNA stability studies revealed an increased half-life of c-*jun* and c-*fos* transcripts resulting from photosensitisation. Although mRNA accumulation after PDT was stronger and more prolonged compared with phorbol ester stimulation, with regard to AP-1 DNA-binding activity, phorbol ester was more efficient. Surprisingly, in addition to the activation of AP-1 DNA-binding via PDT, photodynamic treatment can decrease AP-1 DNA-binding of other strong inducers, such as the protein kinase C-mediated pathway of phorbol esters and the antioxidant pyrrolidine dithiocarbamate (PDTC). This study demonstrates a strong induction of c-*jun* and c-*fos* expression by PDT, with prolonged kinetics and mRNA stabilisation as compared with activation by phorbol esters. Interestingly, this observation is not coincident with an overinduction of AP-1 DNA-binding, hence suggesting that post-translational modifications are dominant regulatory mechanisms after PDT that tightly control AP-1 activity in the nucleus thus limiting the risk of deregulated oncogene expression.

**Keywords:** photodynamic therapy; photosensitisation; oxidative stress; proto-oncogene

Photodynamic therapy (PDT) is a novel therapeutic approach consisting of topical or systemic application of a photosensitising agent and activation by subsequent illumination with tissue-penetrating visible light. The combination of drug uptake in malignant tissue and selective light delivery provides an effective tumour therapy with efficient cytotoxicity and minimal damage to normal tissue (Pass, 1993).

Oxidative stress caused by singlet oxygen ($^1O_2$) is responsible for the effects induced by PDT (Weishaupt *et al.*, 1976). Photosensitisation activates expression of a variety of stress-response genes coding for heat shock proteins, glucose-regulated proteins and haem oxygenase (Gomer *et al.*, 1991; Ryter *et al.*, 1991). The exact functions of these stress proteins are still unclear; however, they might participate in a protective reaction to oxidative stress. Evans *et al.* (1990) demonstrated the dose-dependent production of murine tumour necrosis factor (TNF) in supernatants obtained from PDT-treated macrophages *in vitro*. Furthermore, interleukin (IL) 6 (Kick *et al.*, 1995) and IL-1α/β (G Kick *et al.*, manuscript submitted) protein synthesis induced by PDT in epithelial cell lines was recently discovered. These cytokines have the ability to up-regulate anti-tumour responses and may be good candidates to mediate the local inflammatory reaction. In studying molecular mechanisms of PDT-induced IL-6 expression at the level of gene regulation, electrophoretic mobility shift assays (EMSAs) revealed increased AP-1 DNA-binding activity at the distal AP-1 element of the IL-6 promoter (Kick *et al.*, 1995).

To elucidate further the underlying regulation, the present study analysed the effect of PDT on the AP-1 transcription factor genes c-*jun* and c-*fos*, which are known as proto-oncogenes. Both genes are members of a multigene family coding for transcription factors (Curran and Franza, 1988; Ryder *et al.*, 1989). By interaction of their leucine zipper domain, Jun and Fos proteins form dimeric complexes (Sassone-Corsi *et al.*, 1988; Landschulz *et al.*, 1988; Kouzarides and Ziff, 1988; Gentz *et al.*, 1989). This mode of dimerisation is a characteristic feature of the bZIP family, which includes Jun-, Fos-, activating transcription factor (ATF), cAMP-responsive element-binding (CREB) proteins and others that can bind to the AP-1 DNA motifs and closely related sequence elements (Ivashkiv *et al.*, 1990; Hai and Curran, 1991; Kerppola and Curran, 1993; Hsu *et al.*, 1994; Kataoka *et al.*, 1995), and are regulated by phosphorylation and redox changes (Abate *et al.*, 1990; Boyle *et al.*, 1991; Hunter and Karin, 1992; Dérijard *et al.*, 1994; Kallunki *et al.*, 1994; Kyriakis *et al.*, 1994). AP-1 DNA-binding is considered to be a crucial event in the transcriptional regulation of a variety of genes associated with growth, differentiation, cellular stress and tumorigenesis (Angel and Karin, 1991; Morgan and Curran, 1991; Holbrook and Fornace, 1991; Saez *et al.*, 1995).

Studying the regulation of this ubiquitous transcription

Correspondence: G Messer
Received 13 November 1995; revised 26 January 1996; accepted 29 January 1996

factor in response to singlet oxygen was a further reason for the present investigation, as *in vitro* photosensitisation by Photofrin is a system that generates this reactive oxygen intermediate (Gollnick, 1968; Weishaupt *et al.*, 1976).

Moreover, proto-oncogene expression could be a relevant aspect of cancer therapy. Recent studies describe c-*jun* and c-*fos* expression as associated with programmed cell death (Smeyne *et al.*, 1993; Manome *et al.*, 1993; Kim and Beck, 1994), multidrug-resistant phenotype (Scanlon *et al.*, 1994), and deregulated expression has been discussed as a prerequisite of their transforming potential (Miller *et al.*, 1984; Bos *et al.*, 1990; Lee *et al.*, 1988; van den Berg *et al.*, 1993).

### Materials and methods

### *Cell line and culture conditions*

The human epithelial carcinoma cell line HeLa was grown in adherent culture in Dulbecco's modified Eagle medium (DMEM), supplemented with 10% heat complement-inactivated fetal calf serum (FCS) and 1% penicillin/streptomycin (all purchased from Gibco-BRL, Eggenstein, Germany). For the experiments, HeLa cells were removed by trypsinisation, then washed with phosphate-buffered saline (PBS) and transferred to 5 cm Petri dishes containing medium with only 0.5% FCS. Cells were cultured in a humidified atmosphere at 37°C and 5% carbon dioxide.

### *Reagents*

Pyrrolidine dithiocarbamate (PDTC) was dissolved in PBS as a 1 M stock solution. *N*-acetyl-L-cysteine (NAC) was dissolved in water and adjusted to pH 7.4 by the addition of sodium hydroxide. Both solutions were prepared immediately before use. PDTC, NAC, 12-*O*-tetradecanoyl-phorbol-13-acetate (TPA), cycloheximide (CHX) and actinomycin D (act-D) were purchased from Sigma (Deisenhofen, Germany). Photofrin (Cyanamid Lederle, Wolfratshausen, Germany) was aliquoted at 2.5 mg $ml^{-1}$ stock solution and stored at −20°C.

### *Photodynamic treatment*

Photofrin, a haematoporphyrin derivative preparation, was used as a photosensitiser. HeLa cells were incubated with Photofrin 1 h before illumination, appropriate for an almost complete Photofrin uptake (Leunig *et al.*, 1994). Light application on the monolayers was performed via a 600 $\mu m$ optical fibre by an argon-pumped dye laser (Aesculap Meditech, Heroldsberg, Germany) at a wavelength of 630 nm. This light application system was modified to maintain a homogeneous power density throughout the diameter of the plate. Power density and homogeneity of illumination were continuously controlled by a calibrated power meter (Coherent, Frankfurt am Main, Germany). At a constant power density of 40 mW $cm^{-2}$ up to 400 s, thermal effects upon illumination could be excluded. As determined 24 h after PDT by the trypan blue exclusion method, viability of cells was higher than 80% at all concentrations of Photofrin used up to 2 $\mu g$ $ml^{-1}$ and treated by a light dose of 4 J $cm^{-2}$ as well as under co-treatment.

### *RNA isolation and Northern blot analysis*

Northern blot analyses were performed for detection of specific c-*jun* and c-*fos* transcripts. Hybridisation with a glyceralaldehyde-3-phosphate dehydrogenase (GAPDH) probe was carried out for relative quantification of mRNA levels. Total cellular RNA was isolated by the guanidine isothiocyanate method and acid phenol extraction (Chomczynski and Sacchi, 1987). Cellular RNA (20 $\mu g$) was size-fractionated by electrophoresis using 1.5% denaturing formaldehyde–agarose gels, then transferred to Hybond $N^+$ nylon membranes (Amersham Buchler, Braunschweig, Germany) by capillary blotting. Filters were sequentially hybridised with probes encompassing 1.1 kb of the mouse c-*jun* gene, 1.4 kb of the mouse c-*fos* gene and 210 bp of the human GAPDH gene; all probes were radiolabelled with [$\alpha$-$^{32}$P]dATP (Hartmann Analytic, Braunschweig, Germany) by random hexamer priming (Feinberg and Vogelstein, 1983). Three $\times 10^6$ c.p.m. of labelled probe was used per ml of hybridisation solution [1% bovine serum albumin (BSA), 1 mM disodium EDTA, 250 mM disodium hydrogen phosphate heptahydrate, 7% sodium dodecyl sulphate (SDS); all purchased from Sigma]. Hybridisation was performed at 65°C for 12 h in glass tubes by continuous rotation. Membranes were washed twice at 65°C for 10 min each in a wash buffer (1 mM disodium EDTA, 250 mM disodium hydrogen phosphate heptahydrate, 1% SDS; all purchased from Sigma) to remove unspecifically bound nucleotides. Using amplifying screens, blots were exposed to Kodak X-OMAT AR films (Linhardt, Munich, Germany) at −80°C for up to 2 days.

### *Preparation of cell extracts*

Whole cell extracts were prepared from $5 \times 10^6$ cells. Monolayer cultures were washed twice with ice-cold PBS and scraped from the plates with a rubber spatula. Harvested cells were centrifuged for 10 min at 500 ***g*** at 4°C. Following a single wash with ice-cold PBS, cleared cells were resuspended in 50 $\mu l$ of lysis buffer [20 mM Hepes potassium hydroxide (KOH), pH 7.5, 350 mM potassium chloride, 1 mM magnesium chloride, 0.5 mM EDTA, 0.1 mM EGTA, 5 mM dithiothreitol (DTT), 10 $\mu g$ $ml^{-1}$ leupeptin, 10 $\mu g$ $ml^{-1}$ aprotinin, 0.5% (v/v) of a saturated phenylmethylsulphonyl fluoride (PMSF) solution in ethanol, 20% (v/v) glycerol and 1% (v/v) Nonidet P 40]. After a 15 min incubation on ice, the lysate was centrifuged in a precooled microfuge for 20 min at 14 000 r.p.m. The resulting supernatant was diluted with one volume of buffer H [20 mM Hepes/potassium hydroxide pH 7.9, 0.2 mM EDTA and 20% (v/v) glycerol] to reduce salt concentration (all reagents purchased from Sigma). For all samples, the amount of protein was determined by a Bradford microassay procedure (BioRad, Munich, Germany).

### *Electrophoretic mobility shift assay*

In order to detect the DNA-binding activity of transcription factor AP-1, we used double-stranded (ds) DNA oligonucleotides that encompassed the palindromic AP-1 consensus sequence 5′-TTCCGGCTGACTCATCAAGCG-3′ (purchased from Promega, Heidelberg, Germany). Oligonucleotides were labelled via T4 polynucleotide kinase and [$\gamma$-$^{32}$P]ATP (Hartmann Analytic, Braunschweig, Germany) phosphorylating free 5′-hydroxy groups. DNA protein-binding reactions were carried out in a 20 $\mu l$ reaction volume containing 1–3 $\mu l$ of whole cell extracts (3 $\mu g$ of protein). DNA-binding was initiated by the addition of protein extracts to a mix containing 1–2.5 $\mu g$ of poly (dI–dC), 2 $\mu g$ BSA as a carrier, 2 $\mu l$ of a $10 \times$ binding buffer [100 mM Tris-HCl, pH 7.5, 500 mM sodium chloride, 10 mM EDTA, 50% (v/v) glycerol, 2 mM DTT, 2 $\mu l$ 1% (v/v) Nonidet P 40] (Sigma), and approximately 10 000 c.p.m. of a $\gamma$-$^{32}$P-labelled dsDNA probe with a specific activity not less than 3000 Ci $mmol^{-1}$. After 30 min at room temperature, the samples were loaded on a 4% non-denaturing polyacrylamide gel in $0.5 \times$ Tris–borate–EDTA buffer. Electrophoresis was performed in a vertical EMSA system (AGS, Heidelberg, Germany) at 15 V $cm^{-1}$ for 1.5 h. Gels were dried at 80°C for 30 min and then exposed for autoradiography.

### Results

Our previous study identified the DNA binding of transcription factor AP-1 as an important regulatory event of PDT-induced IL-6 gene expression (Kick *et al.*, 1995). The present investigation proposed to elucidate the regulation of the AP-1 transcription factor genes c-*jun* and c-*fos* by photodynamic treatment. The same cell line and photosensitisation conditions were tested that were previously proven to be efficient in the activation of IL-6 gene expression and AP-1 DNA-binding.

Thus, for the first set of experiments, HeLa cells ($5 \times 10^6$) were incubated with Photofrin at a concentration of $1\ \mu g\ ml^{-1}$ for 1 h, then illuminated with monochromatic (630 nm) light at a dose of $4\ J\ cm^{-2}$. Total RNA was harvested at different points in time following irradiation. The response to phorbol ester TPA ($100\ ng\ ml^{-1}$) that induces comparable levels of IL-6 mRNA and protein (Kick *et al.*, 1995) and c-*jun* and c-*fos* via the protein kinase C pathway was determined as a reference. Thirty minutes after photodynamic treatment, mRNA levels of c-*jun* were already significantly increased, reaching a plateau of maximum levels after 1 to 4 h (Figure 1). Up-regulation of c-*jun* mRNA by PDT was much stronger than by TPA stimulation. In contrast TPA treatment led to a stronger, but more transient induction of c-*fos* mRNA expression. Surprisingly, activation of both proto-oncogenes was clearly prolonged following photodynamic treatment and was still detectable after 8 h. Accumulation of c-*jun* and c-*fos* mRNA was dose-dependent and led to maximum amounts of transcripts at the photosensitiser concentration and illumination conditions used above for kinetics studies. Specific mRNA signals could not be detected in untreated cells or following treatment with either Photofrin or laser light alone (data not shown).

In order to determine whether the up-regulation of c-*jun* and c-*fos* mRNA levels is caused by induction of transcription, HeLa cells were incubated for 2 h with the transcriptional inhibitor actinomycin D ($10\ \mu g\ ml^{-1}$) before PDT. As shown in Figure 2, PDT could not induce c-*jun* or c-*fos* mRNA significantly in cells without associated transcriptional activity (lane 3). To define whether the stability of c-*jun* and c-*fos* mRNA is altered by PDT, HeLa cells were treated with TPA or PDT, then incubated with actinomycin D (act-D) 1 h after activation to stop further transcription. Cells were harvested immediately after the addition of actinomycin D (Figure 2, lanes 4 and 9) and 30 min, 1 h, 2 h and 4 h thereafter. In TPA-treated cells, c-*jun* and c-*fos* mRNA decreased, then disappeared 1 h after adding actinomycin D (Figure 2, lanes 4 to 8). By comparison, following PDT both transcripts showed signals up to 4 h after the addition of actinomycin D (Figure 2, lanes 9 to 13). This result demonstrates that PDT-mediated induction of c-*jun* and c-*fos* mRNA is regulated by the activation of transcription and a prolongation of the mRNA half-life.

To study further whether PDT-induced c-*jun* and c-*fos* mRNA expression is dependent on *de novo* protein synthesis, HeLa cells were treated with PDT in both the presence and absence of cycloheximide, an inhibitor of translation. At concentrations that efficiently inhibit protein synthesis, cycloheximide led to a superinduction of c-*jun* and c-*fos* mRNA (Figure 3, lane 4) with about the same high amounts as upon stimulation by the combination of PDT and cycloheximide (lane 8). Thus, conclusions concerning the necessity of *de novo* protein synthesis could not be drawn from this experiment. However, the early initiation of c-*jun* and c-*fos* mRNA induction by PDT within 30 min indirectly indicates that *de novo* protein synthesis of transcription factors is not involved in the initial step.

Since photodynamic action is caused by oxidative stress, we were prompted to explore the influence of antioxidants on PDT-mediated mRNA uptake. Particularly pyrrolidine dithiocarbamate (PDTC) and *N*-acetyl-L-cysteine (NAC) were tested in multiple studies for efficient radical scavenging at non-cytotoxic concentrations (Schreck *et al.*, 1992; Aruoma *et al.*, 1989; Burgunder *et al.*, 1989). In our *in vitro* system, PDTC (100 $\mu$M) led to induction of c-*jun* and c-*fos* mRNA expression (Figure 3, lane 2). This finding concurs with a previous study that found AP-1 active under both oxidant and antioxidant conditions (Meyer *et al.*, 1993). After exposure to NAC (30 mM), only very weak signals were detectable (Figure 3, lane 3), but both antioxidants preferentially induced c-*fos* transcripts. Interestingly, c-*jun* mRNA induction upon PDT was reduced by PDTC and NAC, and for the latter c-*jun* was selectively suppressed (lanes 5 to 7). Thus, it appeared that c-*jun* expression is mainly caused by the oxidative potential of PDT and could be regarded as an oxidative stress-responsive member of the AP-1 transcription factor family. However, c-*fos* appeared to be much more strongly activated at the mRNA level under antioxidant conditions.

In order to analyse if the strong and prolonged expression of c-*jun* and c-*fos* mRNA by PDT is paralleled by an increased AP-1 DNA-binding activity, protein extracts were prepared at various points in time after photodynamic treatment. Again, TPA stimulation was used as a control. Nuclear extracts were incubated with a $^{32}$P-labelled dsDNA oligonucleotide encompassing the AP-1 consensus sequence. For a comparison at the level of AP-1-binding activity, mobility shift assays were carried out with equal amounts of extracted proteins. An increased AP-1 DNA-binding activity could be detected as early as 15 min following PDT or phorbol ester stimulation (data not shown), and strongest activity was observed after 4 h (Figure 4). At that point in time, AP-1 protein was barely detectable in extracts of untreated HeLa cells (Figure 4, lane 1), but the binding was

**Figure 1** Kinetics of c-*fos* and c-*jun* mRNA induction in HeLa cells by PDT in comparison with stimulation by TPA. Cells ($5 \times 10^6$) were treated with $1\ \mu g\ ml^{-1}$ Photofrin and illuminated with monochromatic light of 630 nm ($4\ J\ cm^{-2}$) or incubated with $100\ ng\ ml^{-1}$ TPA for mitogenic stimulation. The HeLa cells were harvested at the indicated points in time (in h) after treatment. C-*jun* and c-*fos* mRNA was analysed by hybridisation with specific cDNA probes. For relative quantification of mRNA levels, hybridisation with a GAPDH-specific probe was performed after complete stripping of the probes used previously. An autoradiogram is shown.

**Figure 2** C-*jun* and c-*fos* mRNA accumulation by PDT owing to transcriptional activation (lanes 1–3) and mRNA stabilisation (lanes 4–13). Total RNA from HeLa cells was isolated 1 h after PDT with $1\,\mu g\,ml^{-1}$ Photofrin and $4\,J\,cm^{-2}$ (lanes 2 and 3). Incubation with actinomycin D (act-D; $10\,\mu g\,ml^{-1}$) 2 h before photosensitisation (lane 3), and of actinomycin D (act-D; $10\,\mu g\,ml^{-1}$) 1 h after treatment by TPA ($100\,ng\,ml^{-1}$; lanes 4–8) or PDT (lanes 9–13) was performed. Total RNA was isolated immediately (0), 0.5, 1, 2 and 4 h after the addition of actinomycin D. The results for the specific mRNA signals of c-*jun*, c-*fos* and GAPDH as quantitative control are depicted.

**Figure 3** Effects of antioxidant treatment and inhibition of protein synthesis on the induction of c-*jun* and c-*fos* mRNA expression by PDT in HeLa cells. For antioxidant treatment, PDTC was applied at a concentration of $100\,\mu M$ and NAC at a concentration of 30 mM. Inhibition of protein synthesis was induced by cycloheximide ($10\,\mu g\,ml^{-1}$). For co-incubation experiments, PDTC and NAC were added 1.5 h and cycloheximide 0.5 h before light application. Total cellular RNA of HeLa cells was isolated 1 h after PDT ($1\,\mu g\,ml^{-1}$ Photofrin; $4\,J\,cm^{-2}$) and analysed as described in Figure 1.

**Figure 4** Induction of AP-1 DNA-binding in HeLa cells. For electrophorectic mobility shift assay (EMSA), a radioactive labelled dsDNA oligonucleotide comprising the AP-1 canonical consensus sequence motif was incubated with equal amounts of protein extracts from HeLa cells. Cells ($5 \times 10^6$) were treated by PDT ($1\,\mu g\,ml^{-1}$ Photofrin; light: $4\,J\,cm^{-2}$ and 630 nm), TPA ($100\,ng\,ml^{-1}$), PDTC ($100\,\mu M$) or kept in medium without further treatment (Co). Co-stimulation with TPA was performed 15 min after (PDT + TPA) or before (TPA + PDT) photodynamic treatment. PDTC was added 1.5 h before illumination. The fluorogram of a native gel is shown. The position of the protein DNA complex containing AP-1 is indicated with a filled arrowhead, whereas the free radiolabelled oligonucleotide is marked with an open triangle. Exposition of the autoradiogram was performed for 15 h at −80°C with an intensifier screen.

strongly induced by TPA stimulation (lane 3). The same photodynamic treatment (Photofrin: $1\,\mu g\,ml^{-1}$; light: $4\,J\,cm^{-2}$) that led to c-*jun* and c-*fos* mRNA expression resulted in activation of AP-1 DNA-binding (Figure 4, lane 2). Despite the relatively strong and prolonged c-*jun* and c-*fos* mRNA induction upon PDT, stimulation by TPA was more efficient in inducing AP-1 activities at the level of DNA-binding.

The antioxidant PDTC was shown to activate transcription factor AP-1 at the transcriptional and post-translational level (Meyer *et al.*, 1993). Accordingly, we detected an increased AP-1 DNA-binding activity following antioxidant treatment (Figure 4, lane 6). Interestingly, the strong AP-1 activity upon PDTC addition was markedly reduced if antioxidant treatment was combined with photodynamic treatment (Figure 4, lanes 6 and 7) at conditions that otherwise independently led to AP-1 activation (lane 2).

The DNA-binding activity induced by TPA was also reduced if phorbol ester stimulation was combined with photodynamic treatment. The addition of TPA 15 min after (Figure 4, lane 4) and 15 min before (lane 5) photodynamic treatment resulted in a reduced binding activity as compared with TPA stimulation alone (lane 3).

### Discussion

In this study, photodynamic treatment of human epithelial HeLa cells led to rapid and strong induction of c-*jun* and c-*fos* mRNA expression. Both genes were induced by similar kinetics, but c-*jun* was much more responsive to PDT. Striking differences were apparent when kinetics of c-*jun* and c-*fos* gene induction by PDT and TPA were compared. The prolonged induction of both nuclear oncogenes by photodynamic treatment was in sharp contrast to their transient activation by the phorbol ester TPA. These results suggest a specific regulatory modus of immediate early genes by Photofrin-mediated photosensitisation, predominantly generating singlet oxygen. Other reactive oxygen intermediates, such as superoxide anions (Crawford *et al.*, 1988; Shibanuma

*et al*., 1988; Amstad *et al*., 1992) and hydrogen peroxide (Nose *et al*., 1991; Devary *et al*., 1991), activate c-*jun* and c-*fos* transcripts by way of similar kinetics with rapid induction and immediate down-regulation of mRNA signals, just as described for the immediate–early gene response to serum or TPA stimulation. One recent study examined the effects of photosensitiser-mediated oxidative stress on the activation of c-*jun* and c-*fos* mRNA (Luna *et al*., 1994). Their results obtained from RIF-1 cells differ from our human system. The authors received already increased mRNA levels of c-*fos* and c-*jun* at the time of illumination. The kinetics of gene activation, which they found to be dependent on PKC, were similar to the TPA or serum response. This could be explained by the exchange of the medium with increasing concentrations of FCS 30 min before (5–10%) and after (15%) illumination (Luna *et al*., 1994; Treisman, 1986).

Regulation of c-*jun* and c-*fos* expression after photodynamic treatment alone occurred at both the transcriptional and post-transcriptional levels. Our mRNA stability studies clearly demonstrated increased half-life of induced c-*jun* and c-*fos* transcripts after PDT. Thus, mRNA stabilisation of proto-oncogenes by singlet oxygen might account for the strong and prolonged induction of c-*jun* and c-*fos*.

Degradation of mRNA is described as an important step of c-*jun* and c-*fos* gene regulation, and is responsible for rapid restriction of protein synthesis. The turnover of c-*fos* mRNA depends on two instability domains, the first located within the 3′ untranslated region of the mRNA and consisting of an AU-rich sequence element (Shyu *et al*., 1989; Chen and Shyu, 1994). The second domain is positioned in the transcribed sequence and referred to as the coding region determinant of mRNA instability of the c-*fos* gene (Kabnick and Housman, 1988; Chen *et al*., 1992). At the same time, AT-rich sequences of the untranslated region of c-*jun* may be involved in the post-transcriptional regulation of the c-*jun* gene (Hattori *et al*., 1988). Oxidative stress resulting from photosensitisation might interfere with protein behaviour at the described destabilising sites, with unspecific mRNA-degrading activities or transcriptional elongation blocks (Plet *et al*., 1995).

Regarding the fundamental importance of AP-1 *vis á vis* cell differentiation and proliferation, it seems likely that aberrant regulation of any component involved might have dramatic consequences, such as eventual loss of growth control and neoplastic transformation. Is deregulated c-*jun* and c-*fos* mRNA expression a prerequisite for neoplastic transformation by photodynamic therapy? Although there is evidence for a causal relationship between mRNA stability and transformation potential, oncogenic activation of c-*jun* or c-*fos* requires both deregulated expression and structural changes. To activate the transforming potential of c-*fos*, a 67 bp element in the 3′ non-coding region which leads to mRNA destabilisation must be deleted (Miller *et al*., 1984; Lee *et al*., 1988; Meijlink *et al*., 1985; Rahmsdorf *et al*., 1987). Tumour induction by high-level c-*fos* expression has been observed in an animal model; c-*fos* transgenic mice were constructed under the control of the metallothionein promoter and the FBJ 3′ LTR, which was necessary to stabilise the exogenous *fos* mRNA (Rüther *et al*., 1989).

Furthermore, it appears insufficient to evaluate the risk of carcinogenesis by AP-1 at the level of mRNA expression, since there are several post-translational regulatory mechanisms and, moreover, synergistic or antagonistic interactions between AP-1 family members and possibly other transcription factors.

In the case of photodynamic treatment, post-translational regulation also appears to be a powerful mechanism in controlling AP-1 activity. Although mRNA accumulation by PDT was stronger and even prolonged in comparison with phorbol ester stimulation, on the level of protein DNA-binding, photodynamic treatment was much less efficient. Apparently, the binding capacity of AP-1 is determined by regulatory mechanisms other than transcriptional activation of the nuclear factor genes. According to previous reports, AP-1 was shown to be induced under both pro-oxidant and anti-oxidant conditions (Abate *et al*., 1990; Xanthoudakis *et al*., 1992). Stronger DNA-binding was caused by the anti-oxidant state (Meyer *et al*., 1993; Schenk *et al*., 1994; Li and Jaiswal, 1994). Superoxide anions and hydrogen peroxide were found to be weak inducers of AP-1 binding (Amstad *et al*., 1992; Devary *et al*., 1991). Reversible oxidation of a single, conserved cysteine residue located in the DNA-binding domain of AP-1 proteins modulates their DNA-binding activity (Abate *et al*., 1990). Oxidation of these cysteine sites inhibits the DNA-binding activity of AP-1 (Abate *et al*., 1990; Bannister *et al*., 1991; Frame *et al*., 1991). Ref-1 (redox factor 1), a DNA repair protein, was found to reactivate *in vitro* oxidised AP-1 by reducing this cysteine residue (Xanthoudakis *et al*., 1992). Singlet oxygen-mediated redox regulation of AP-1 binding could therefore explain the discrepancy between strong mRNA induction and weak DNA-binding of AP-1 after PDT. Furthermore, oxidation of cysteine residues might be responsible for the down-regulation of AP-1 DNA-binding induced by the antioxidant PDTC upon concomitant photodynamic treatment.

As phorbol ester-mediated AP-1 DNA activation is caused by dephosphorylation (Boyle *et al*., 1991; Papavassiliou *et al*., 1992) and PKC seems not to be involved in PDT-mediated regulation of AP-1 in HeLa cells (Kick *et al*., 1995), redox regulation might be regarded as the dominant pathway of AP-1 DNA-binding modulated upon PDT. This argument is strengthened by the fact that activation of AP-1 upon TPA was suppressed by PDT both before and after the addition of the phorbol ester. However, there is growing evidence for AP-1 regulating cytokine gene expression in response to PDT.

This research has identified Photofrin-mediated photosensitisation as a potent inducer of c-*jun* and c-*fos* mRNA expression but a comparatively weak activator of AP-1 DNA-binding. Mechanisms of PDT-mediated AP-1 regulation differ from mechanisms of activation by other reactive oxygen intermediates and phorbol esters, thus suggesting a unique regulatory pathway. It seems unlikely that deregulated mRNA expression could enhance the potential risk of PDT-induced carcinogenesis, since AP-1 DNA-binding is tightly controlled.

**Acknowledgements**

This investigation was supported by a grant from the Bundesministerium für Forschung und Technologie (0706903A5 to AG) and the Dr Bodo Sponholz Stiftung (Frankfurt, Germany). The authors thank Professor K Messmer for his continuous support and making it possible to perform part of the experiments at his Institute for Surgical Research, Klinikum Groβhadern, Ludwigen-Maximillians University of Munich, and Dr P Schliesser for helpful comments on the manuscript.

### References

ABATE C, PATEL L, RAUSCHER FJ AND CURRAN T. (1990). Redoxregulation of fos and jun DNA-binding activity *in vitro*. *Science*, **249**, 1157–1161.

AMSTAD PA, KRUPITZA G AND CERUTTI PA. (1992). Mechanism of c-fos induction by active oxygen. *Cancer Res*., **52**, 3952–3960.

ANGEL P AND KARIN M. (1991). The role of Jun, Fos and the AP-1 complex in cell-proliferation and transformation. *Biochim. Biophys. Acta*, **1072**, 129–157.

ARUOMA OI, HALLIWELL B, HOEY BM AND BUTLER J. (1989). The antioxidant action of N-acetylcysteine: its reaction with hydrogen peroxide, hydroxyl radical, superoxide, and hypochlorus acid. *Free Radical Biol. Med.*, **6**, 593–597.

BANNISTER AJ, COOK A AND KOUZARIDES T. (1991). *In vitro* DNA binding activity of Fos/Jun and BZLF1 but not C/EBP is affected by redox changes. *Oncogene*, **6**, 1243–1250.

BOS TJ, MONTECLARO FS, MITSUNOBO F, BALL AR, CHANG CHW, NISHIMURA T AND VOGT PK. (1990). Efficient transformation of chicken embryo fibroblasts by c-jun requires structural modification in coding and noncoding sequences. *Genes Dev.*, **4**, 1677–1687.

BOYLE WJ, SMEAL T, DEFIZE LHK, ANGEL P, WOODGETT R, KARIN M AND HUNTER T. (1991). Activation of protein kinase C decreases phosphorylation of c-Jun at sites that negatively regulate its DNA binding activity. *Cell*, **64**, 573–584.

BURGUNDER JM, VARRIALE A AND LAUTERBURG BH. (1989). Effect of N-acetylcysteine on plasma cysteine and glutathione following paracetamol administration. *Eur. J. Clin. Pharmacol.*, **36**, 127–131.

CHEN CA, YOU Y AND SHYU AB. (1992). Two cellular proteins bind specifically to a purine-rich sequence necessary for the destabilization function of a c-fos protein-coding region determinant of mRNA instability. *Mol. Cell. Biol.*, **12**, 5748–5757.

CHEN CY AND SHYU AB. (1994). Selective degradation of early-response gene mRNAs: functional analyses of sequence features of the AU-rich elements. *Mol. Cell. Biol.*, **14**, 8471–8482.

CHOMCZYNSKI P AND SACCHI N. (1987). Single-step method of RNA isolation by acid guanidinium thiocyanate–phenol–chloroform extraction. *Anal. Biochem.*, **162**, 156–159.

CRAWFORD D, ZBINDEN I, AMSTAD P AND CERUTTI PA. (1988). Oxidant stress induces the proto-oncogenes c-fos and c-myc in mouse epidermal cells. *Oncogene*, **3**, 27–32.

CURRAN T AND FRANZA BR. (1988). Fos and Jun: the AP-1 connection. *Cell*, **55**, 395–397.

DERIJARD B, HIBI M, WU IH, BARRETT T, SU B, DENG T, KARIN M AND DAVIS RJ. (1994). JNK1: A protein kinase stimulated by UV light and ha-ras that binds and phosphorylates the c-Jun activation domain. *Cell*, **76**, 1025–1037.

DEVARY Y, GOTTLIEB RA, LAU LF AND KARIN M. (1991). Rapid and preferential activation of the c-jun gene during the mammalian UV-response. *Mol. Cell. Biol.*, **11**, 2804–2811.

EVANS S, MATTHEWS W, PERRY R, FRAKER D, NORTON J AND PASS HI. (1990). Effect of photodynamic therapy on tumor necrosis factor production by murine macrophages. *J. Natl Cancer Inst.*, **82**, 34–39.

FEINBERG AP AND VOGELSTEIN B. (1983). A technique for radiolabeling DNA restriction endonuclease fragments to high specific activity. *Anal. Biochem.*, **132**, 6–13.

FRAME MC, WILKIE NM, DARLING AJ, CHUDLEIGH A, PINTZAS A, LANG JC AND GILLESPIE DAF. (1991). Regulation of AP-1/DNA complex formation *in vitro*. *Oncogene*, **6**, 205–209.

GENTZ R, RAUSCHER FJ, ABATE C AND CURRAN T. (1989). Parallel association of Fos and Jun leucine zippers juxtaposes DNA binding domains. *Science*, **243**, 1695–1699.

GOLLNICK K. (1968). Type II photooxygenation reactions in solution. *Adv. Photochem.*, **6**, 1–122.

GOMER CJ, LUNA M, FERRARIO A AND RUCKER N. (1991). Increased transcription and translation of heme oxygenase in Chinese hamster fibroblasts following photodynamic stress of Photofrin II incubation. *Photochem. Photobiol.*, **53**, 275–279.

HAI T AND CURRAN T. (1991). Cross-family dimerization of transcription factors Fos/Jun and ATF/CREB alters DNA binding specificity. *Proc. Natl Acad. Sci. USA*, **88**, 3720–3724.

HATTORI K, ANGEL P, LE BEAU MM AND KARIN M. (1988). Structure and chromosomal localization of the functional intronless human jun protooncogene. *Proc. Natl Acad. Sci. USA*, **85**, 9148–9152.

HOLBROOK NJ AND FORNACE AJ. (1991). Response to adversity: molecular control of gene activation following genotoxic stress. *New. Biol.*, **3**, 825–833.

HSU W, KERPPOLA TK, CHEN PL, CURRAN T AND CHEN-KIANG S. (1994). Fos and Jun repress transcription activation by NF-IL6 through association at the basic zipper region. *Mol. Cell. Biol.*, **14**, 268–276.

HUNTER T AND KARIN M. (1992). The regulation of transcription by phosphorylation. *Cell*, **70**, 375–387.

IVASHKIV LB, LIOU HC, KARA CJ, LAMPH WW, VERMA IM AND GLIMCHER LH. (1990). mXBP/CRE-BP2 and c-jun form a complex which binds to the cyclic AMP, but not to the 12-*O*-tetradecanoylphorbol-13-acetate, response element. *Mol. Cell. Biol.*, **10**, 1609–1621.

KABNICK KS AND HOUSMAN DE. (1988). Determinants that contribute to cytoplasmatic stability of human c-fos and $\beta$-globin mRNAs are located at several sites in each mRNA. *Mol. Cell. Biol.*, **8**, 3244–3250.

KALLUNKI T, SU B, TSIGELNY I, SLUSS HK, DERIJARD B, MOORE G, DAVIS R AND KARIN M. (1994). JNK2 contains a specificity-determining region responsible for efficient c-Jun binding and phosphorylation. *Genes Dev.*, **8**, 2996–3007.

KATAOKA K, IGARASHI K, ITOH K, FUJIWARA T, NODA M, YAMAMOTO M AND NISHIZAWA M. (1995). Small maf proteins heterodimerize with fos and may act as competitive repressors of the NF-E2 transcription factor. *Mol. Cell. Biol.*, **15**, 2180–2190.

KERPPOLA TK AND CURRAN T. (1993). Selective DNA binding by a variety of bZIP proteins. *Mol. Cell. Biol.*, **13**, 5479–5489.

KICK G, MESSER G, GOETZ A, PLEWIG G AND KIND P. (1995). Photodynamic therapy induces expression of interleukin 6 by activation of AP-1 but not NF-$\kappa$B DNA binding. *Cancer Res.*, **55**, 2373–2379.

KIM R AND BECK WT. (1994). Differences between drug-sensitive and -resistant human leukemic CEM cells in c-jun expression, AP-1 DNA-binding activity, and formation of Jun/Fos family dimers, and their association with internucleosomal DNA ladders after treatment with VM-26. *Cancer Res.*, **54**, 4958–4966.

KOUZARIDES T AND ZIFF E. (1988). The role of the leucine zipper in the fos–jun interaction. *Nature*, **336**, 646–651.

KYRIAKIS JM, BANERJEE P, NIKOLAKAKI E, DAL T, RUBIE EA, AHMAD MF, AVRUCH J AND WOODGETT JR. (1994). The stress-activated protein kinase subfamily of c-Jun kinases. *Nature*, **369**, 156–160.

LANDSCHULZ WH, JOHNSON PF AND MCKNIGHT SL. (1988). The leucine zipper: a hypothetical structure common to a new class of DNA binding proteins. *Science*, **240**, 1759–1764.

LEE WM, LIN C AND CURRAN T. (1988). Activation of the transforming potential of the human fos proto-oncogene requires message stabilization and results in increased amounts of partially modified fos protein. *Mol. Cell. Biol.*, **8**, 5521–5527.

LEUNIG A, STAUB F, PETERS J, HEIMANN A, CSAPO C, KEMPSKI O AND GOETZ AE. (1994). Relation of early photofrin uptake to photodynamically induced phototoxicity and changes of cell volume in different cell lines. *Eur. J. Cancer*, **30**, 78–83.

LI Y AND JAISWAL AK. (1994). Human antioxidant-response–element-mediated regulation of type 1 NAD(P)H:quinone oxidoreductase gene expression. *Eur. J. Biochem.*, **226**, 31–39.

LUNA MC, WONG S AND GOMER CJ. (1994). Photodynamic therapy mediated induction of early response genes. *Cancer Res.*, **54**, 1374–1380.

MANOME Y, DATTA R, TANEJA N, SHAFMAN T, BUMP E, HASS R, WEICHSELBAUM R AND KUFE D. (1993). Coinduction of c-jun gene expression and internucleosomal DNA fragmentation by ionizing irradiation. *Biochemistry*, **32**, 10607–10613.

MEIJLINK F, CURRAN T, MILLER AD AND VERMA IM. (1985). Removing of a 67-basepair sequence in the noncoding region of protooncogene fos converts it to a transforming gene. *Proc. Natl Acad. Sci. USA*, **82**, 4987–4991.

MEYER M, SCHRECK R AND BAEUERLE PA. (1993). $H_2O_2$ and antioxidants have opposite effects on activation of NF-kappa B and AP-1 in intact cells: AP-1 as secondary antioxidant-responsive factor. *EMBO J.*, **12**, 2005–2015.

MILLER AD, CURRAN T AND VERMA IM. (1984). C-fos protein can induce cellular transformation: a novel mechanism of activation of a cellular oncogene. *Cell*, **36**, 51–60.

MORGAN JI AND CURRAN T. (1991). Stimulus-transcription coupling in the nervous system: involvement of the inducible proto-oncogenes fos and jun. *Annu. Rev. Neurosci.*, **14**, 421–451.

NOSE K, SHIBANUMA M, KIKUCHI K, KAGEYAMA H, SAKIYAMA S AND KUROKI T. (1991). Transcriptional activation of early-response genes by hydrogen peroxide in a mouse osteoblastic cell line. *Eur. J. Biochem.*, **201**, 99–106.

PAPAVASSILIOU AG, BOHMANN K AND BOHMANN D. (1992). Determining the effect of inducible protein phosphorylation on the DNA-binding activity of transcription factors. *Anal. Biochem.*, **203**, 302–309.

PASS HI. (1993). Photodynamic therapy in oncology: mechanisms and clinical use. *J. Natl Cancer Inst.*, **85**, 443–456.

PLET A, EICK D AND BLANCHARD JM. (1995). Elongation and premature termination of transcripts initiated from c-fos and c-myc promoters show dissimilar patterns. *Oncogene*, **10**, 319–328.

RAHMSDORF HJ, SCHÖNTHAL A, ANGEL P, LITFIN M, RÜTHER U AND HERRLICH P. (1987). Posttranslational regulation of c-fos mRNA expression. *Nucleic Acids Res.*, **15**, 1643–1659.

RÜTHER U, KOMITOWSKI D, SCHUBERT FR AND WAGNER EF. (1989). C-fos expression induces bone tumors in transgenic mice. *Oncogene*, **4**, 861–865.

RYDER K, LANAHAN A AND PEREZ-ALBUERNE E. (1989). *Jun* D: a third member of the *jun* gene family. *Proc. Natl Acad. Sci. USA*, **86**, 1500–1503.

RYTER SW, FERRARIO A, FISHER AM, LUNA M, RUCKER N, WONG S AND GOMER CJ. (1991). Photooxidative stress and photodynamic therapy. In *Oxidative Damage and Repair, Chemical, Biological and Medical Aspects*, Davies KJA. (ed.) pp. 300–310. Pergamon: Oxford.

SAEZ E, RUTBERG SE, MUELLER E, OPPENHEIM H, SMOLUK J, YUSPA SH AND SPIEGELMAN BM. (1995). C-fos is required for malignant progression of skin tumors. *Cell*, **82**, 721–732.

SASSONE-CORSI P, RANSONE LJ, LAMPH WW AND VERMA ID. (1988). Direct interaction between fos and jun nuclear oncoproteins: role of the 'leucine zipper' domain. *Nature*, **336**, 692–695.

SCANLON KJ, ISHIDA H AND KASHANI-SABET M. (1994). Ribozyme-mediated reversal of the multidrug-resistant phenotype. *Proc. Natl Acad. Sci. USA*, **91**, 11123–11127.

SCHENK H, KLEIN M, ERDBRÜGGER W, DRÖGE W AND SCHULZE-OSTHOFF K. (1994). Distinct effects of thioredoxin and antioxidants on the activation of transcription factors NF-κB and AP-1. *Proc. Natl Acad. Sci. USA*, **91**, 1672–1676.

SCHRECK R, MEIER B, MÄNNEL DN, DRÖGE W AND BAEUERLE PA. (1992). Dithiocarbamates as potent inhibitors of nuclear factor κB activation in intact cells. *J. Exp. Med.*, **175**, 1181–1194.

SHIBANUMA M, KUROKI T AND NOSE K. (1988). Induction of DNA replication and expression of proto-oncogene c-*myc* and c-*fos* in quiescent Balb/3T3 cells by xanthine/xanthine oxidase. *Oncogene*, **3**, 17–21.

SHYU AB, GREENBERG ME AND BELASCO JG. (1989). The c-fos transcript is targeted for rapid decay by two distinct mRNA degradation pathways. *Genes Dev.*, **3**, 60–72.

SMEYNE RJ, VENDRELL M, HAYWARD M, BAKER SJ, MIAO GG, SCHILLING K, ROBERTSON LM, CURRAN T AND MORGAN JI. (1993). Continuous c-fos expression precedes programmed cell death *in vivo*. *Nature*, **363**, 166–169.

TREISMAN R. (1986). Identification of a protein-binding site that mediates transcriptional response of the c-*fos* gene to serum factors. *Cell*, **46**, 567–573.

VAN DEN BERG S, RAHMSDORF HJ, HERRLICH P AND KAINA B. (1993). Overexpression of c-fos increases recombination frequency in human osteosarcoma cells. *Carcinogenesis*, **14**, 925–928.

WEISHAUPT KR, GOMER CJ AND DOUGHERTY T. (1976). Identification of singlet oxygen as the cytotoxic agent in photoinactivation of a murine tumor. *Cancer Res.*, **36**, 2326–2329.

XANTHOUDAKIS S, MIAO G, WANG F, PAN YCE AND CURRAN T. (1992). Redox activation of Fos–Jun DNA binding activity is mediated by a DNA repair enzyme. *EMBO J.*, **11**, 3323–3335.